# POCUS - a new computer-based training approach for improving the quality of ultrasound diagnostics in gallbladder diseases

**DOI:** 10.1186/s12909-026-09336-y

**Published:** 2026-05-01

**Authors:** Florian Recker, Stefan Michel, Manuela Lehmann, Gebhard Mathis, Joseph Osterwalder

**Affiliations:** 1 Department for Obstetrics and Prenatal Medicine, University Hospital Bonn, Venusberg Campus 1, 53127 Bonn, Germany; 2 School for Applied Psychology (APS), University of Applied Sciences and Arts Northwestern Switzerland (FHNW), Olten, Switzerland; 3 Dr. Summer-Strasse 3, Rankweil, AT-6830 Austria; 4 Polipraxis AG, Marktgasse 3, St. Gallen, 9000 Switzerland

**Keywords:** Point-of-Care Ultrasound (POCUS), Image Recognition and Interpretation Training, Competence Assessment, Medical Education, Self-Assessment

## Abstract

**Purpose:**

Point-of-care ultrasound (POCUS) has gained widespread adoption in medical diagnostics due to its simplicity, accessibility, and cost-effectiveness. However, insufficient training remains a significant challenge and limits its effective use. Particularly neglected is image pattern recognition and interpretation. Conventional educational methods are struggling to meet the growing demand for comprehensive POCUS training. This study aims to address this gap by introducing a novel approach using a computer-based image recognition and interpretation training (CBIRIT).

**Materials and methods:**

In a prospective randomized controlled study, 46 medical students were divided into three groups: conventional teaching alone, conventional teaching with supplementary CBIRIT, and a control group with no training. A competency assessment test measured diagnostic performance in gallbladder disease detection. Pre- and post-test results were analyzed using non-parametric tests to compare performance within and between groups.

**Results:**

The CBIRIT group showed a significant improvement in diagnostic performance (*p* < .001). In contrast, the conventional teaching group showed no significant improvement. Interestingly, this group exhibited increased confidence (*p* < .05) without improved performance, suggesting overconfidence.

**Conclusion:**

CBIRIT significantly improves diagnostic performance in POCUS when compared to traditional teaching methods. It offers a resource-efficient solution to POCUS training, addressing conventional methods’ limitations and reducing overconfidence in diagnostic judgments. This approach also supports skill assessment and recertification.

## Introduction

Point-of-care ultrasound (POCUS) is a diagnostic ultrasound and monitoring exam or intervention performed and interpreted at bedside by the attending physician as part of a standard patient-physician encounter [1]. However, the scope of the examination – from simple yes-no questions to comprehensive and complex problems such as for example volume management – depends on the individual situation and expertise of the examiner. The need for training has grown due to the accuracy, efficiency, affordability, and increasing use of mobile diagnostic devices, as well as demand from young doctors to integrate them into practice [1]. Due to its intuitive and simple applicability, there is a high risk that POCUS will be used in an uncontrolled manner without appropriate training, especially on image recognition and interpretation, and thus posing a safety risk [2, 3]. A study demonstrated that a one-day POCUS course significantly improved knowledge, image recognition and interpretation skills, and confidence among novice trainees and attending physicians [4]. However, limited training can lead to overconfidence where participants overestimated their abilities after minimal training [5]. Further studies highlighted that medical residents were more confident but less accurate than experienced physicians, indicating a discrepancy between confidence and competence [6]. Feedback was found to be effective in mitigating overconfidence in image recognition and interpretation [7].

Adequate training is often lacking, especially in image pattern recognition and image interpretation. Conventional methods cannot meet the growing demand due to limited resources and instructors, so efficient training solutions are urgently needed. A promising solution from the security sector has been adapted for POCUS training in image recognition and interpretation. This software is globally used in airport security training for threat detection. Several studies in this field have shown that the use of computer-based image recognition and interpretation training can substantially improve image interpretation performance and thus increase effectiveness and efficiency [8, 9]. Although these results pertain to the interpretation of X-ray images, a similar pattern can be observed in the case of POCUS.

**Fig. 1:**
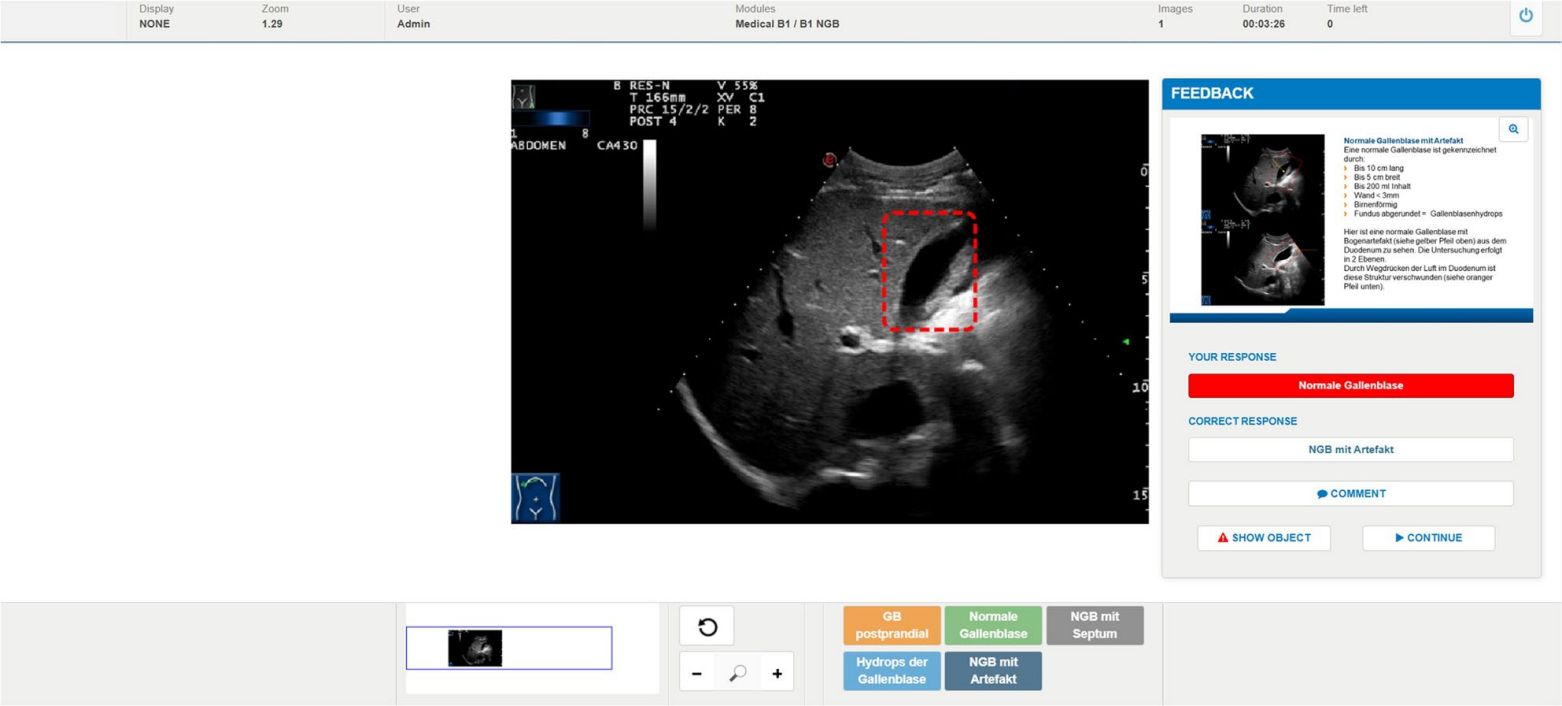
Screenshot of the Computer-based Image Recognition and Interpretation Training (CBIRIT)

Building on this work, we developed and tested a computer-based image recognition and interpretation training (CBIRIT) for POCUS and evaluated its impact on diagnostic accuracy, confidence, and interpretation speed compared to traditional teaching methods and a control group. A screenshot of the CBIRIT can be seen in Fig. 1.

## Methods and materials

**Fig. 2:**
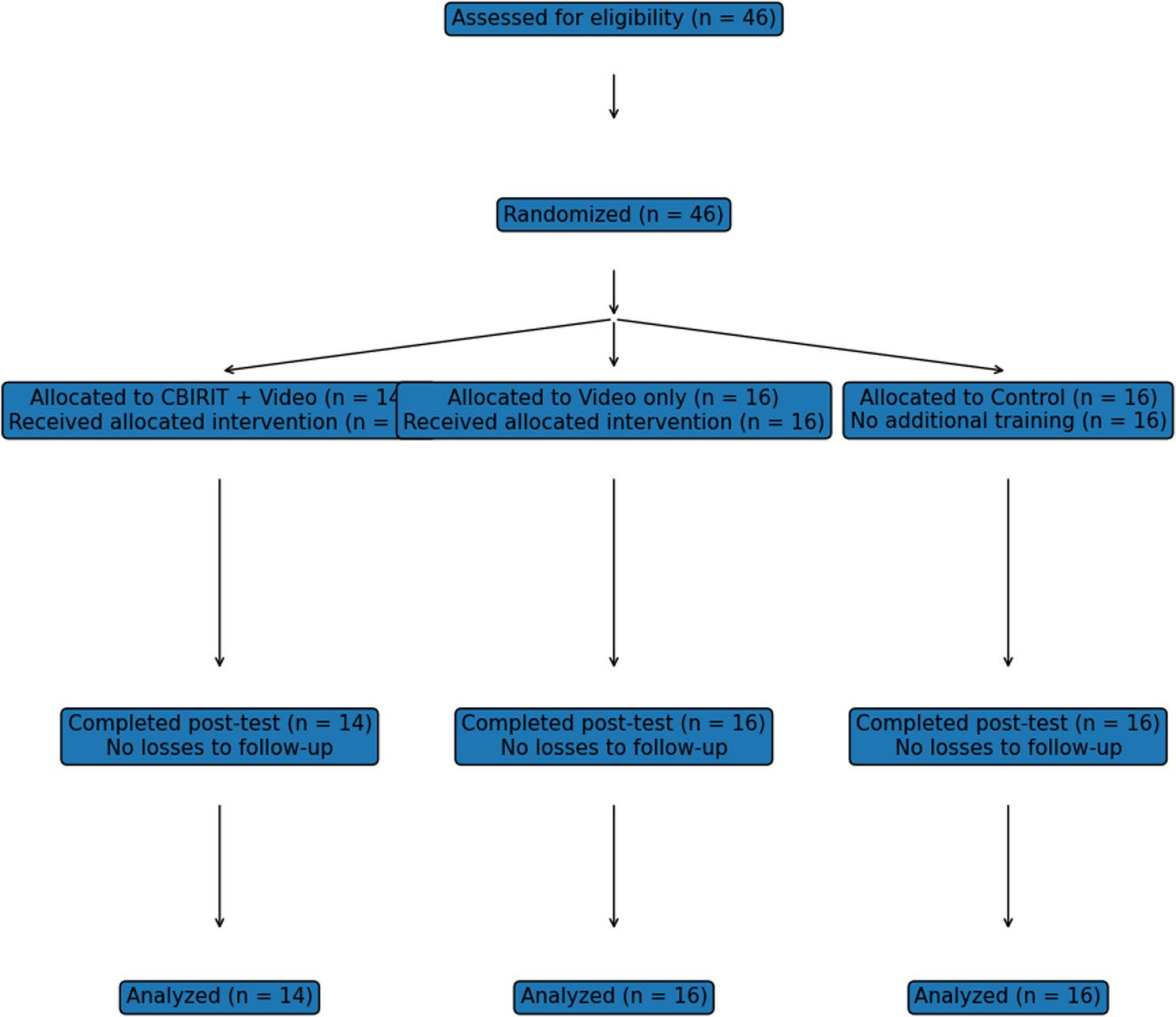
Consent flow diagram

This prospective randomized controlled study recruited medical students without prior POCUS experience via email at a German University. Interested participants received study information, gave informed consent, and completed a demographic questionnaire (age, gender, ultrasound experience). A priori power analysis using G*Power 3.1 indicated that 14 participants were required to detect a large effect (*d_z* = 0.75) with 80% power at α = 0.05 in a one-tailed Wilcoxon signed-rank test (Fig. 2).

All participants received a 32-minute pre-recorded video titled Fundamentals of Sonography, narrated by an ultrasound expert, covering ultrasound applications, frequency ranges, and image interpretation guidelines [10]. Participants confirmed full viewing to ensure standardized baseline knowledge.

Subsequently, they completed the Ultrasound Competency Assessment Test (UCAT) pre-test, consisting of 41 gallbladder ultrasound images representing various pathologies (see Table 1). After four practice trials with feedback, participants interpreted each image within 20 s, selecting a diagnosis from 12 options and rating their confidence on a 5-point Likert scale. No feedback was given during the main test.

**Table 1:** Image content of the Ultrasound Competency Assessment Test (UCAT)

Gallstones with or without sludge (5 images)	Acalculous cholecystitis or other wall thickening (edema, etc.) (3 images)
Cholesterol polyp (3 images)	Normal gallbladder with or without artifacts (4 images)
Adenompolyp (7 images)	Gallbladder postprandial (contracted) (3 images)
Cholesterolosis or Adenomyomatosis (2 images)	Sludge: No sedimentation (3 images)
Acute cholecystitis with gallstones (4 images)	Sludge with sedimentation (1 image)
Chronic stone cholecystitis with gallstones (5 images)	Sludge: Tumorous cluster with or without gallstones (1 image)

Participants were then randomly assigned to three groups (Group 1: *n* = 14, Group 2: *n* = 16, Control: *n* = 16).

Group 1 received two training components: a 29-minute gallbladder anatomy video including ultrasound techniques as well as common errors and CBIRIT with 61 gallbladder cases across six difficulty levels, including feedback and repetition for incorrect responses. Group 2 received The ultrasound images were carefully selected from the personal archives of two recognized experts (GM with more than 50 years and JO with more than 40 years of experience in abdominal sonography). The underlying diagnoses had been clinically confirmed through surgical interventions, further imaging procedures, or the documented clinical course.

For the final selection of the annotated images used in the examination and training module, two half-day workshops were conducted. Both experts participated in these sessions together with an IT specialist. During these workshops, the diagnoses were independently re-evaluated and verified to ensure a high level of diagnostic accuracy and validity.

Only the gallbladder anatomy video. The control group proceeded directly to the post-test without additional training.

All participants then completed the UCAT post-test, identical in format to the pre-test. The entire study was conducted on participants’ personal computers, with standardized technical guidelines provided to ensure consistent display conditions.

Following the testing phase, participants were debriefed and provided with contact information for potential follow-up. All data were anonymized and securely stored for subsequent analysis.

This study is a prospective randomized educational intervention study. Although randomized, it does not constitute a clinical trial under WHO or ICMJE definitions, as it evaluates an educational training intervention without patient-related health outcomes. Therefore, prospective trial registry registration was not required.

**Fig. 3:**
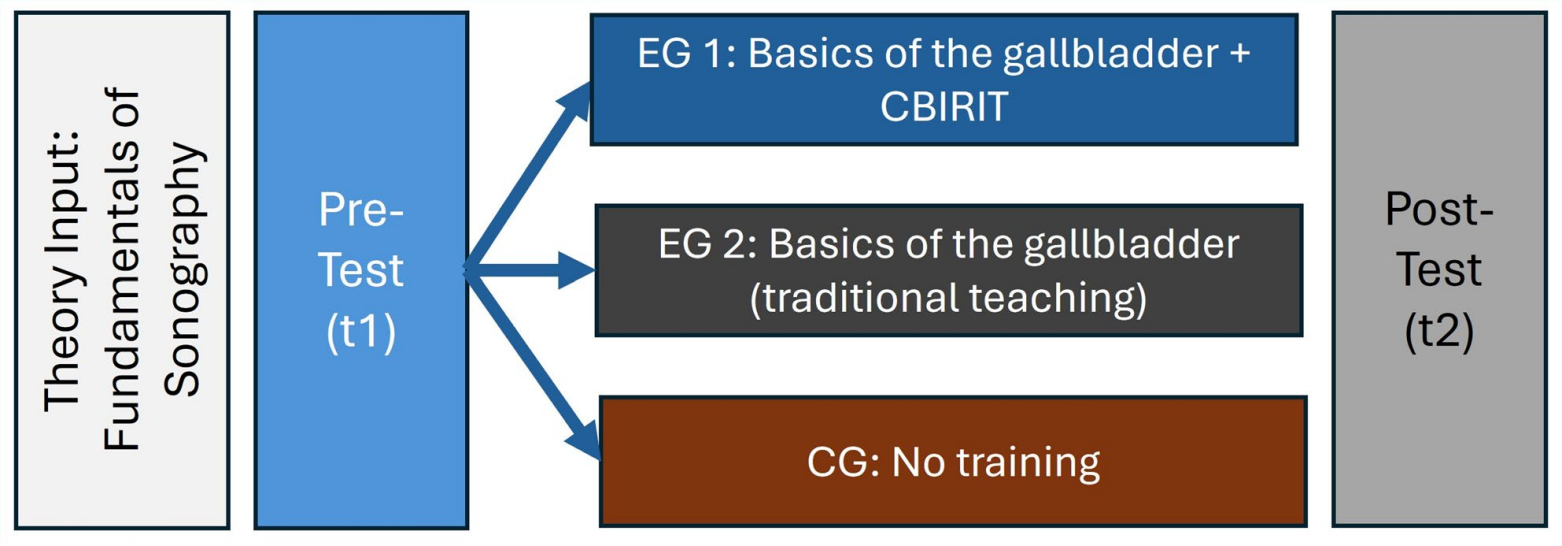
Study design. EG 1: Experimental group 1; EG 2: Ecperimental group 2; CG: Control group

An overview of the study design is presented in Fig. 3.

### Statistical analysis

Statistical analyses were conducted using Jamovi (version 2.3.28). Normal distribution was assessed with the Shapiro-Wilk test. Since only two variables were normally distributed across all groups (post-test % correct answers; post-test confidence rating), the non-parametric Kruskal-Wallis H-test was used for group comparisons. Medians were calculated for test results. The Wilcoxon signed-rank test was used to compare pre- and post-test results within groups. Effect sizes were calculated using the rank-biserial correlation (*r* < sub> rb</sub> ). As no universally accepted benchmarks exist for this measure, values of approximately 0.10, 0.30, and 0.50 were used as rough guidelines for small, medium, and large effects, respectively, following conventions for correlation coefficients [11].

## Results

### Sample characteristics and randomization

A total of 46 medical students from a German university participated in this study (33% male, 67% female; *M* = 24.33, *SD* = 3.09). Participants were randomly assigned to one of three groups (CBIRIT, traditional teaching, control).

Baseline equivalence across groups was confirmed. A one-way analysis of variance (ANOVA) showed no significant differences in pretest scores, *F*(2, 43) = 0.31, *p* = .733. Groups also did not differ significantly in age, *F*(2, 43) = 0.59, *p* = .559, or gender distribution, *F*(2, 43) = 0.55, *p* = .582, indicating successful randomization.

Descriptive characteristics of the groups were as follows: The CBIRIT group (*n* = 14) had a mean age of 23.5 years (*SD* = 3.29) and 79% female participants. The traditional teaching group (*n* = 16) had a mean age of 24.6 years (*SD* = 3.49), with 62.5% female participants. The control group (*n* = 16) had a mean age of 24.6 years (*SD* = 2.12), also with 62.5% female participants.

### Primary outcome: diagnostic performance

**Fig. 4:**
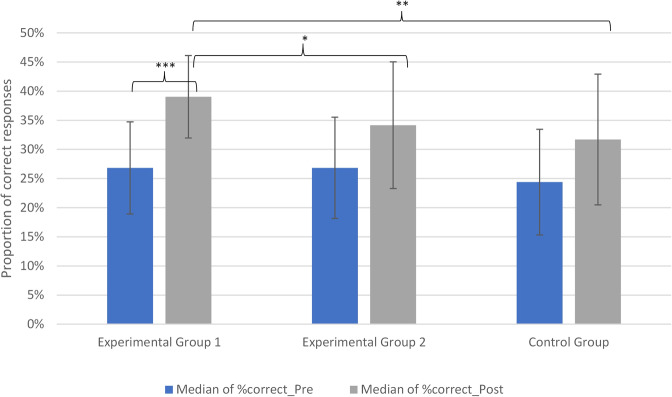
Difference between experimental group 1, experimental group 2 and the control group considering the proportion of correct responses each forthe pre- and post-test. Left bar: Median percentage of correct answers in the pre-test; right bar: median percentage of correct answers in the post-test

The primary outcome of this study was the change in diagnostic accuracy (percentage of correct answers) from pretest to posttest which can be seen in Fig. 4.

In the CBIRIT group, diagnostic accuracy increased from a median of 27% at pretest to 39% at posttest. A Wilcoxon signed-rank test indicated that this improvement was statistically significant, *W* = 3.00, *p* < .001, rank-biserial *r* = .94.

In contrast, the traditional teaching group showed a non-significant increase, *W* = 44.00, *p* = .11, *r* = .35, and no significant change was observed in the control group, *W* = 60.50, *p* = .36, rank-biseria *r* = .11.

Between-group comparisons of posttest performance further supported these findings. Diagnostic accuracy was significantly higher in the CBIRIT group compared to the traditional teaching group (*p* = .040) and the control group (*p* = .003). No significant difference was observed between the traditional teaching and control groups (*p* = .224; see Table 2).

**Table 2:** Results of the post-test comparisons between the groups for % correct answers

Comparison	Medians post-test (%)	U-Value	*p*-Value	Effect Size (rank-biserial *r*)
EG1 vs. EG2	39 vs. 34	70	0.040*	0.375
EG1 vs. CG	39 vs. 32	46.5	0.003**	0.585
EG2 vs. CG	34 vs. 32	108	0.224	0.160

### Secondary outcomes

#### Interpretation times

**Fig. 5:**
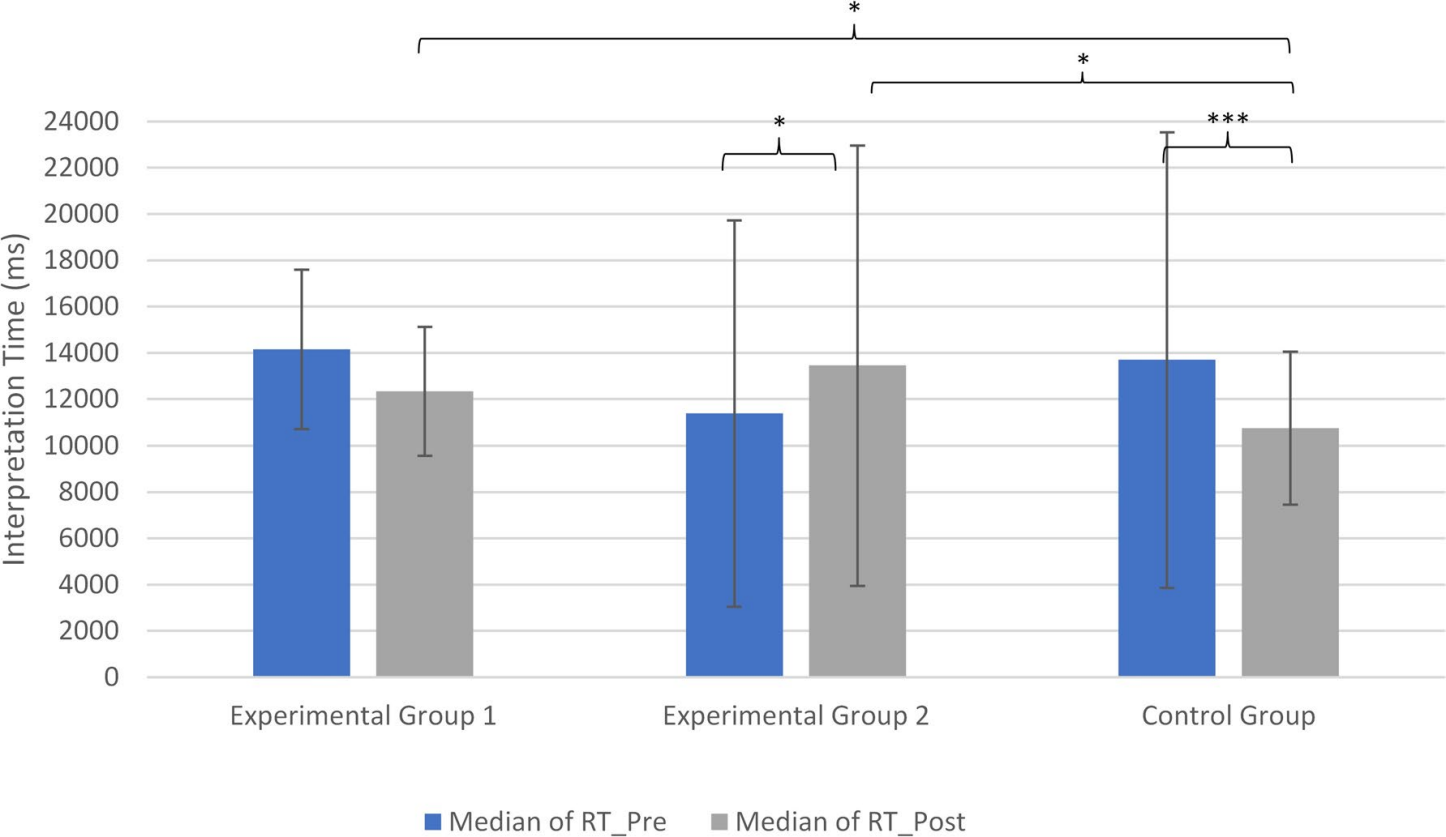
Interpretation time for the three groups for the pre- and post-test. Left bar: median of interpretation time in the pre-test; right bar: median ofinterpretation time in the post-test

Interpretation times were analyzed as a secondary outcome to assess efficiency of diagnostic decision-making. No significant baseline differences between groups were observed which can be seen in Fig. 5.

Within-group analyses indicated that interpretation times in the CBIRIT group decreased numerically from pretest (*Mdn* = 14,153 ms) to posttest (*Mdn* = 12,341 ms), but this change did not reach statistical significance, *W* = 76.00, *p* = .08, rank-biseria *r* = .45.

The traditional teaching group showed a significant increase in interpretation times, from *Mdn* = 11,384 ms to *Mdn* = 13,454 ms, *W* = 33.00, *p* < .05, rank-biseria *r* = .52. In contrast, the control group showed a significant decrease, from *Mdn* = 13,695 ms to *Mdn* = 10,752 ms, *W* = 131.00, *p* < .001, rank-biseria *r* = .93.

Between-group comparisons at posttest revealed significantly shorter interpretation times in the control group compared to both experimental groups (both *p* < .05; see Table 3).

**Table 3:** Results of the post-test comparisons between the groups for interpretation times in milliseconds

Comparison	Medians (ms) post-test	U-Value	*p*-Value	Effect Size (*r*)
EG1 vs. EG2	12’341 vs. 13’454	101	0.667	0.098
EG1 vs. CG	12’341 vs. 10’752	61	0.034*	0.455
EG2 vs. CG	13’454 vs. 10’752	76	0.026*	0.406

These results should be interpreted with caution, as interpretation time was not the primary outcome of the study.

#### Confidence rating

**Fig. 6:**
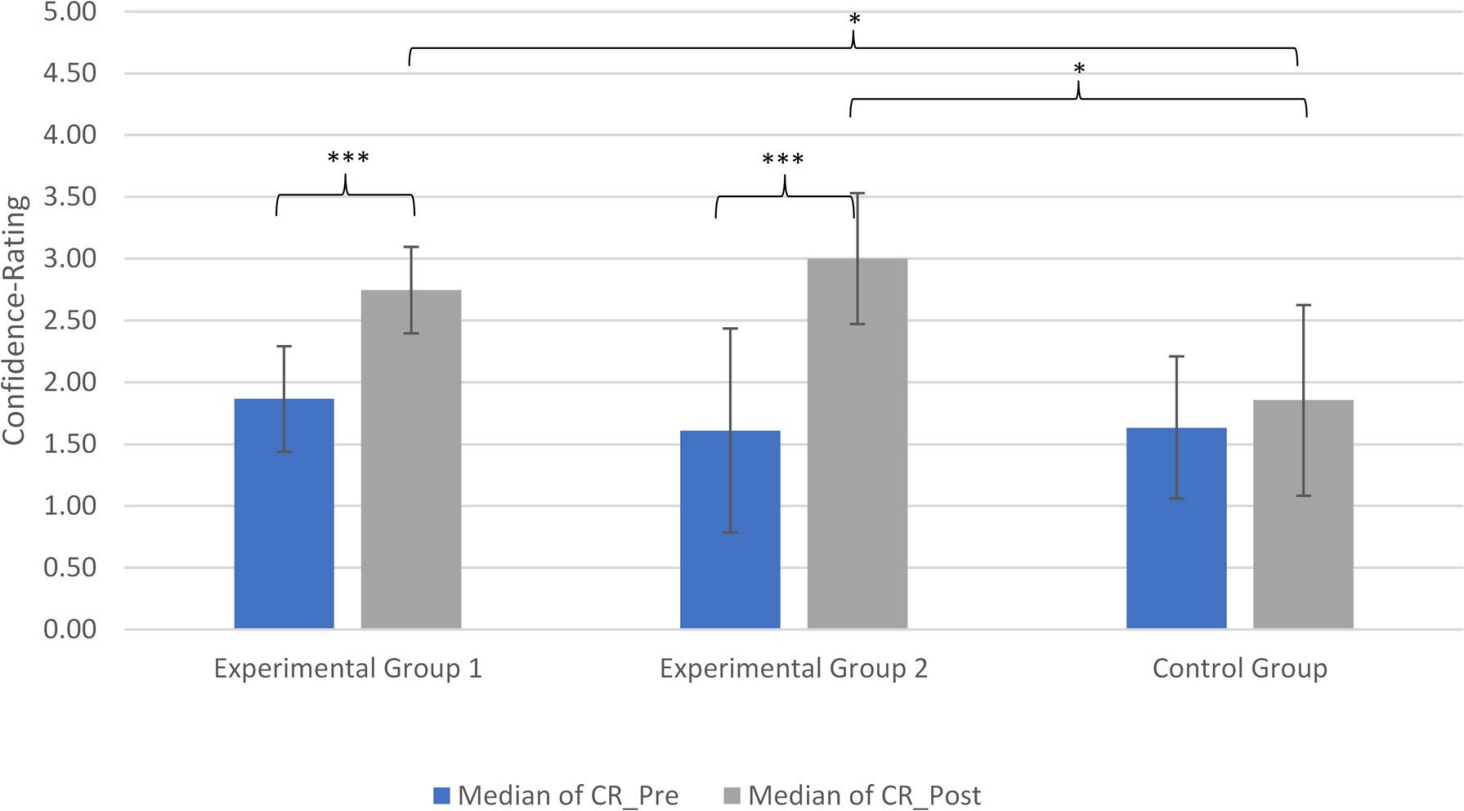
Confidence rating of the three groups for the pre- and post-test. Left bar: median of conficence rating in the pre-test; right bar: median of conficence rating in the the post-test

Confidence ratings were assessed as a secondary outcome using a 5-point Likert scale. No significant baseline differences between groups were observed which can be seen in Figs. 6.

Within-group analyses showed that confidence increased significantly in both experimental groups. In the CBIRIT group, confidence increased from *Mdn* = 1.87 to *Mdn* = 2.74, *W* = 0.00, *p* < .001, *r* = 1.00. This effect size reflects that all participants in this group showed an increase in confidence from pretest to posttest, resulting in a uniform direction of change. In the traditional teaching group, confidence increased from *Mdn* = 1.61 to *Mdn* = 3.00, *W* = 12.00, *p* < .01, rank-biseria *r* = .82.

No significant change was observed in the control group (*W* = 48.00, *p* = .51, rank-biseria *r* = .02).

At posttest, both experimental groups reported significantly higher confidence than the control group (both *p* < .05), while no significant difference was found between the experimental groups (see Table 4).

**Table 4:** Results of the post-test comparisons between the groups for confidence ratings on a 5-point scale

Comparison	Medians post-test	U-Value	*p*-Value	Effect Size (*r*)
EG1 vs. EG2	2.74 vs. 3.00	97	0.546	0.134
EG1 vs. CG	2.74 vs. 1.85	59.5	0.030*	0.469
EG2 vs. CG	3.00 vs. 1.85	68.5	0.026*	0.465

### Exploratory findings

Across conditions, the pattern of results suggests a potential speed–accuracy trade-off, particularly in the control group, where reduced interpretation times were not accompanied by improved diagnostic accuracy. This observation is exploratory and should be interpreted cautiously.

## Discussion

The present study investigated the effectiveness of computer-based image recognition and interpretation training (CBIRIT) for teaching point-of-care ultrasound (POCUS), with a primary focus on diagnostic accuracy and additional secondary outcomes including interpretation time and confidence.

### Primary outcome: diagnostic accuracy

The primary aim of this study was to evaluate whether CBIRIT improves diagnostic accuracy. The results indicate that participants in the CBIRIT group showed a substantial and statistically significant improvement in diagnostic performance from pretest to posttest, whereas no significant improvements were observed in the traditional teaching or control groups.

These findings suggest that CBIRIT may be an effective approach for enhancing diagnostic accuracy in POCUS training. One possible explanation lies in its adaptive, level-based design, which allows learners to engage repeatedly with diagnostic cases while receiving immediate and targeted feedback. This approach is consistent with learning theories emphasizing active engagement, deliberate practice, and feedback as key mechanisms for skill acquisition [12].

In contrast, the absence of significant improvement in the traditional teaching group may indicate that conventional instructional formats are less effective in fostering applied diagnostic skills, at least within the time frame and context of this study. Taken together, the results support the use of interactive and individualized training approaches for developing diagnostic competence. However, given the relatively small sample size, these findings should be interpreted with caution and require replication in larger, ideally preregistered studies.

### Secondary outcomes

#### Interpretation time

Interpretation time was examined as a secondary outcome reflecting efficiency in diagnostic decision-making. The findings across groups showed a heterogeneous pattern.

While the control group demonstrated a significant reduction in interpretation time, this change was not accompanied by improved diagnostic accuracy. In contrast, participants in the CBIRIT group achieved higher diagnostic accuracy without a significant increase or decrease in interpretation time. The traditional teaching group showed an increase in interpretation time.

These results may suggest differences in processing strategies across conditions. The reduction in interpretation time observed in the control group, in the absence of accuracy gains, could reflect a tendency toward faster but less thorough decision-making. Conversely, the relatively stable interpretation times in the CBIRIT group, combined with improved accuracy, may indicate a more structured and deliberate approach to image interpretation.

However, given that interpretation time was not the primary outcome of the study, these findings should be interpreted with caution.

#### Confidence and its relation to performance

Confidence was also analyzed as a secondary outcome. Both experimental groups showed significant increases in self-reported confidence, whereas no significant change was observed in the control group.

Importantly, only the CBIRIT group demonstrated concurrent improvements in both confidence and diagnostic accuracy. In contrast, the traditional teaching group showed increased confidence without a corresponding improvement in performance.

This pattern may indicate a potential mismatch between perceived and actual competence in the traditional teaching condition. However, because no significant improvement in diagnostic accuracy was observed in this group, this interpretation should be considered tentative. Such discrepancies have been discussed in the literature as a possible source of diagnostic error [5–7], but further research is needed to examine this relationship in the present context.

In contrast, the alignment of confidence and performance observed in the CBIRIT group may reflect more accurate self-assessment, potentially facilitated by repeated feedback and structured practice. Nevertheless, these interpretations remain tentative, as confidence was not a primary outcome of the study.

#### Exploratory considerations

Across outcomes, the results suggest a potential speed–accuracy trade-off, particularly in the control group, where faster interpretation times were not associated with improved diagnostic performance. This observation is exploratory and should be interpreted cautiously, as the study was not specifically designed or powered to test this relationship.

#### Implications for POCUS training

The present findings have implications for the design of POCUS training programs. The observed improvements in diagnostic accuracy associated with CBIRIT are consistent with prior research in domains such as radiology and aviation security, where computer-based training has been shown to enhance image interpretation skills [8, 9].

The adaptive and feedback-oriented structure of CBIRIT may offer a scalable approach to training, particularly in contexts where access to expert instruction is limited. However, given that diagnostic accuracy was the only prespecified primary outcome, conclusions regarding broader educational benefits should be drawn with caution.

### Limitations and future directions

Several limitations should be considered when interpreting the findings. First, the study was conducted with medical students, which may limit generalizability to more experienced clinicians. Second, CBIRIT was implemented alongside traditional teaching, making it difficult to isolate its independent effects. Third, the focus on relatively simple gallbladder pathologies may not reflect the complexity of real-world diagnostic scenarios. Finally, the short duration of the intervention precludes conclusions about long-term retention of skills.

Future research should examine the effectiveness of CBIRIT across different clinical domains (e.g., cardiac, vascular, and obstetric imaging) and assess its long-term impact on diagnostic performance and confidence. Additionally, studies with practicing clinicians and more complex cases would help to further establish its applicability in clinical settings.

## Conclusion

This study provides evidence that computer-based image recognition and interpretation training (CBIRIT) can enhance diagnostic accuracy in POCUS training. Beyond its immediate educational benefits, CBIRIT offers a scalable and resource-efficient approach that may help address current gaps in POCUS education, particularly in settings with limited access to expert instruction.

From a clinical perspective, integrating CBIRIT into training curricula may support more consistent development of diagnostic skills and promote safer decision-making in bedside ultrasound. In addition, its standardized and feedback-driven structure suggests potential utility not only for initial training but also for ongoing competency assessment and recertification.

Future research should examine its effectiveness in more complex clinical scenarios and among practicing clinicians to further establish its role in routine medical education and practice.

## Data Availability

The datasets generated and/or analysed during the current study are available from the corresponding author on reasonable request.

## Abbreviations

POCUS: Point-of-care ultrasound
CBIRIT: Computer-based image recognition and interpretation training
UCAT: Ultrasound Competency Assessment Test
